# Treatment of Typhoid Fever

**Published:** 1854-02

**Authors:** C. D. Robinson

**Affiliations:** Almond, N. Y.


### BUFFALO MEDICAL JOURNAL

AND

### MONTHLY REVIEW.

VOL. 9. FEBRUARY, 1854. NO. 9.

### ORIGINAL COMMUNICATIONS.

ART. I.— *Treatment of Typhoid Fever.* By C. D. Robinson, M. D., Almond, N. Y.

At the risk of being thought affected with typhoidism, I propose to give you a short statement of the treatment in the following cases of typhoid fever.

I will premise by saying that I have been in practice twenty years in this region of country, and that the disease appears to be on the increase, decidedly, of late, as few will deny who have paid the least attention to the subject.

Having, with many others, doubted the efficacy of the treatment usually adopted, my mind was directed to the subject by reading Dr. Flint's remarks on the abortive treatment of typhoid fever, in the Buffalo Medical Journal of Feb., 1852, I determined to contrast the different modes of treatment with a view to satisfy myself of their comparative merit.

Case I.—Mrs. O., aged 48, of good constitution. Saw her Oct. 19th; had been sick about a week; had typhoid fever complicated with pneumonia. The typhoid symptoms were well pronounced.

℞ Blister to the chest; opii and quinine; stimulating expectorants, brandy and water.

Continued this mode of treatment about ten days, when convalescence took place. She continued improving until Nov. 7, when she was found dead in her bed, supposed from an old cardiac affection. A post-mortem was no held.

Case II.—N. O., aged 19, son of Mrs. O., was sick when I first visited his mother. Complained of chills, headache, pain in back and limbs, skin dry and hot, tongue slightly coated, pulse about natural.

℞ Cathartic. Adopted the expectant plan of treatment, gave small doses of pulv. Doveri and sup. tart. potassa, liq. ac. ammo. and spts. nitre, &c.

Continued the above plan, with such modification as appeared necessary, in conjunction with small doses of Hyd. C. Creta, and frequent showering the head and sponging the body with cold water, for three weeks, when an apparent amendment took place, but which was soon followed by an aggravation of all the symptoms. Tongue became dry and dark; sordes collected about the mouth; pulse very frequent; low muttering delirium; diarrhœa and tympanitis were present. To these were soon superadded epistaxis to an alarming extent. Simultaneously blood oozed from the entire internal surface of the mouth. The breath and exhalations from the patient were very offensive, having a putrescent smell. Petechiæ appeared on the back and legs, commingled with large discolored patches, having the appearance of interstitial hæmorrhage. He was now put upon the use of remedies calculated to restore energy to the vital powers, as quinine, brandy, mineral acids, chlorate of potassa, &c., and the application of astringents to the bleeding surfaces. Notwithstanding the vigorous use of the above remedies, the bleeding continued for six days, when he began to amend. Convalescence was very protracted, as he required close attention till the last of November.

Case III.—D. O., aged 22, brother of the above, was attacked with chills on the 15th Nov. First visited by Dr. J. W. Black, my partner, on the 16th. He reports his condition as follows: Patient complains of intense headache; pulse 120; great thirst; urine scanty and high colored; tongue covered with a thin coat, a dark streak through the center; face and eyes suffused; skin dry and hot; feels stupid and inclines to doze.

℞ Oleum Ricine.

Visited him again in the evening. The oil had moved his bowels freely.

℞ Sulph. quinine, gr. xx.
Tr. opii, gutt. xxx.

To be taken at once, in peppermint-water, followed next morning by liq. ac. ammo., three parts and spts. nitre one part — a tablespoonful every two hours.

*Nov.* 17. Pulse 120; skin moist, otherwise the same.

℞ Sulph. quinine, ʒss.

Tr. opii, gutt. xl.

Taken at bedtime. Continue the mixture as above.

*Nov.* 18. Passed a good night. Sweat freely; skin soft, and tongue moist and cleaned in patches; pulse 96, full and soft; face and eyes suffused; complains of dizziness and ringing in the ears; less headache than the day before; urine free; complains of oppression of the thorax; slight expectoration of bloody mucus.

℞ Sulph. quinine, ʒss.

Tr. opii, ʒj. in peppermint water.

Continued lig. ac. ammo. and spts. nitre as before.

*Nov.* 19. Reports better; passed a good night; sweat profusely; complains of feeling weak; pulse 92; tongue cleaning at the edges; roaring in the head which he compares to the sound of a grist-mill; skin soft; urine free; bowels open.

℞ Sulph. quinine, gr. xx.

Tr. opii, ʒj., taken at bedtime.

Continue the spts. min. and nitre as above.

*Nov.* 20. Pulse 100; complains of pain in the left side, extending to iliac region; expectorates frothy mucus tinged with blood.

℞ Sinapism to the affected side. Pulv. Doveri, hyd. C. creta and pulv-glyc., every three hours. Mucil. gum Arab.

*Nov.* 21. Looks better; pulse 85; tongue clean; skin moist. Passed a good night, and says he would feel well but for the pain in his side.

℞ Enema. A powder of ipecac., gr. j. Hyd. C. creta., gr. ij. Opii, gr. ss. Every four hours. Blister to the side.

*Nov.* 22. Expresses himself as feeling decidedly better. Bowels not having moved, an ounce of castor oil was taken, which had procured three or four evacuations. Says he has sweat considerable through the night. Cough less severe, expectorates no blood; tongue moist.

From this time he began slowly to improve and was discharged about the fifteenth day. Since then we have heard but little of him until about four or five days ago, when he came into the office complaining of cough and shortness of breath, and œdema of the lower extremities. Since then he has expectorated a large quantity of purulent matter, evidently showing the existence of an abscess in the left lung, for which he is now under treatment.

Case IV.—L. O'C., aged 25, was attacked, Oct. 26th, with the usual symptoms of typhoid fever of mild character.

℞ Cathartic of castor oil, followed by a powder of quinine, gr. v., sulph. mor., gr. ss., ipecac, gr. j., every six hours, alternated with spts. nitre and liq. ac. ammo.

This treatment was continued four or five days, with an occasional exhibition of a cathartic. The patient not improving we fell back upon the expectant method. This fever continued twenty-one days, when he began to amend. Convalescence has been very protracted, as phlebitis of one of the lower extremities followed, attended with great debility.

Case V.—Miss E. H., aged 17, was attacked with the usual symptoms of typhoid fever. Saw her first Nov. 8th; had been sick four days.

℞ Cathartic, followed by opii, gr. iv., *at once.*

*Nov.* 9. Feels better; sweat freely through the night; tongue moist and losing its redness.

℞ Opii, gr. ss.
Ipecac, gr. ¼.
Pulv. gum Arabic, gr. iv., in powder, every six hours, alternated with a weak solution of super. carb. soda.

She was discharged on the eighth day, perfectly recovered, and was soon able to engage in her daily avocations.

Case VI.—J. W., aged 20, was attacked on the 20th Nov. with chills, headache, thirst, pain in back and limbs, and other symptoms denoting typhoid fever. Dr. Black visited him on the morning of the 21st.

℞ An ounce of castor oil, to be repeated, if necessary, followed by opii, gr. v., liq. ac. ammo. and spts. nitre, equal parts—a tablespoonful every two hours through the day.

Saw him again at 9 o'clock, P. M. Found him sweating; tongue moist: pulse reduced from 100 to 90; complains of pain in the chest; hacking cough; less headache, and has slept some through the day.

℞ Repeat the opium at 11 o'clock. Continue liq. ac. ammo. and spts. nitre, as before.

*Nov.* 22. Passed a good night; had two copious evacuations from the bowels, two hours after taking the opium; says he feels weak and prostrated; skin soft; tongue moist; urine free; pulse 108, small and weak.

℞ Sulph. quinine, gr. ij.
Carb. ammo., gr. ij., and
Pulv. glyc. gr. ij. every four hours, and occasionally a little Port wine and water.

*Nov.* 23. Bowels open; complains of pain in the right side; pulse 108, soft and compressible; skin moist; urine free.

℞ Ipecac. gr. ½, opii, gr. j., and super. carb. soda, gr. ij., every four hours, alternated with spts. nitre, combined with syrup of squill and senega.

*Nov.* 24. Appears better this morning; pulse 96, and full; tongue moist and cleaning; slight cough; free from pain; countenance looks decidedly better. From this time convalescence may be dated, as he continued rapidly to improve, and was discharged on the 29th.

Case VII.—Mrs. B., aged 48, was attacked with typhoid fever, complicated with pneumonitis. Saw her first on the 14th Nov.; had been sick three days. As the pneumonia symptoms were urgent, bled her from the arm, and prescribed cathartic, followed by a solution of tart. emetic and pulv. Doveri, alternately every two hours. Continued the above treatment until typhoid symptoms began to show themselves, when she was put upon the use of opium and quinine, stimulating expectorants, and Port wine, when an amendment soon took place, and was followed by complete convalescence about the fifteenth day.

Case VIII.—Miss C., aged 20. First visited her on the 23d Nov. Found her with the usual symptoms of the prevailing fever.

℞ Cathartic, followed by opii, grs. iv.

*Nov.* 24. Improved.

℞ Opii, gr. j., and pulv. gum Arabic, grs. iv., every six hours, alternated with liq. ac. ammo. and spts. nitre.

Continued the opium treatment for a day or two, and discharged her convalescent on the tenth day.

Being at a neighboring village on business, I visited, in company with her attending physician, a young lady just attacked with typhoid fever, which was then prevailing in that place to a considerable extent; by my advice he gave her opii, gr. iv., followed by the plan of treatment as above stated.

I have since learned from him that all symptoms of fever disappeared in the course of four or five days, as was the case in one or two other patients to whom the opium was administered.

L. S., aged 20, was attacked with symptoms of the prevailing fever; saw him on the second day.

℞ Castor oil, followed at bedtime by opii, grs. v.

All traces of fever disappeared during the next twenty-four hours, and in a day or two he resumed his usual occupation.

Perhaps our experiments do not sufficiently establish the fact that opium may be regarded as a specific in typhoid fever, yet I am satisfied it is a remedy of *great* power, frequently arresting the disease in the onset, and in all the cases in which it was given, exerted a marked influence, materially shortening its career and rendering it more manageable. Convalescence after its use, was rapid, and recovery more complete, than is usual after the other modes of treatment. When given early in the disease, it appears to arrest its progress, by destroying the virus upon which it depends. If not given until the disease is fully established and the system completely imbued with the poison, it appears to shield the vital powers from its effect. Perhaps its operation, in this respect, may be somewhat analogous to that of chloroform in protecting the system from the shock and prostration of a surgical operation.

Our experience with the quinine was not as satisfactory, although, perhaps, we failed to give it a sufficient trial. Case 3d, in which we adopted the heroic treatment recommended by Dr. Fenner, of New Orleans, the fever was evidently arrested in the course of four or five days, but symptoms of pneumonitis soon supervened. Could the large quantity of quinine given him have had any agency in producing the thoracic inflammation?

As regards the usual modes of treatment in this disease, I am satisfied that, except when complicated with inflammation of a vital organ which cannot be considered as an element of the disease, blood-letting is positively injurious,—the same remark applies to purgatives and the alterative treatment with mercury, as it exerts no control whatever over typhoid fever, and, indeed, as is the case with purgatives, it tends to aggravate the follicular inflammation and the pre-existing irritation of the mucous surface of the intestinal canal, always present. But whatever may be the opinion of the profession in regard to opium and quinine as remedies in typhoid fever, certainly great praise is due to Drs. Henry, of Ill., and Fenner, of New Orleans, for their efforts to find a remedy for this disease, the treatment of which, hitherto, has been justly considered among the *opprobria medicorum.*

